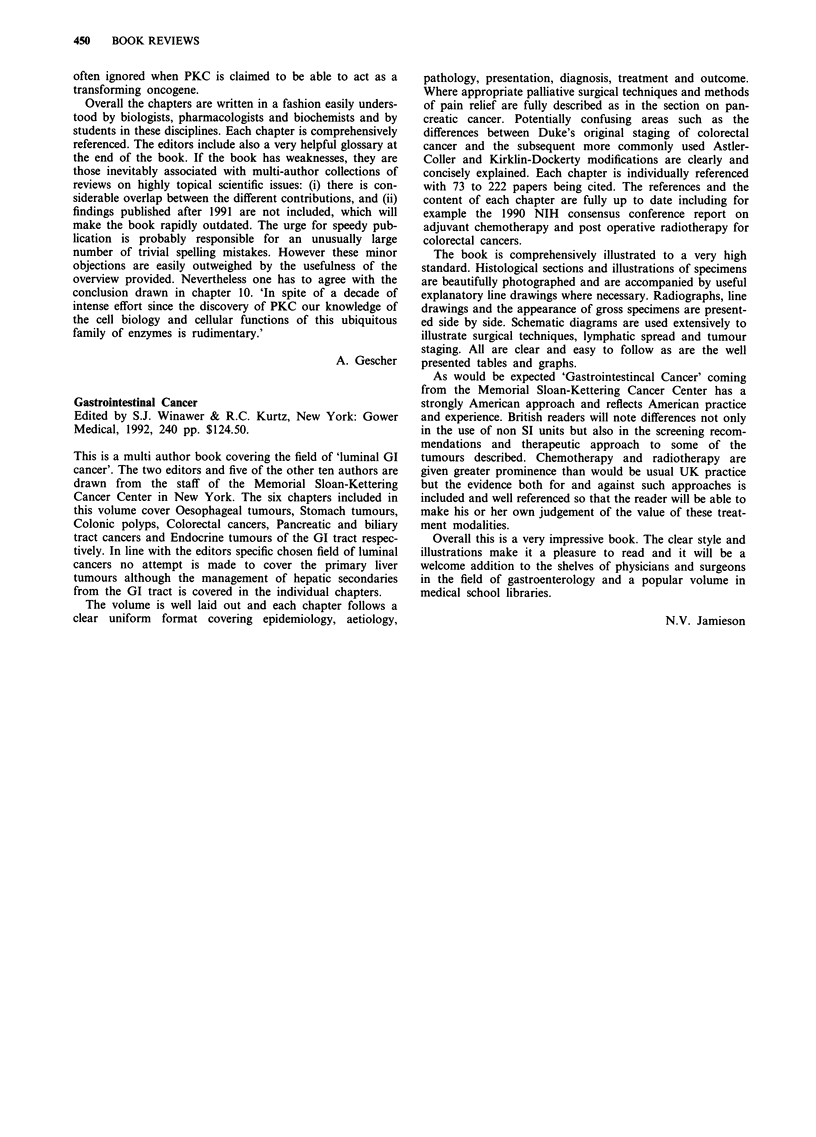# Gastrointestinal Cancer

**Published:** 1993-08

**Authors:** N.V. Jamieson


**Gastrointestinal Cancer**

Edited by S.J. Winawer & R.C. Kurtz, New York: Gower Medical, 1992, 240 pp. $124.50.

This is a multi author book covering the field of 'luminal GI cancer'. The two editors and five of the other ten authors are drawn from the staff of the Memorial Sloan-Kettering Cancer Center in New York. The six chapters included in this volume cover Oesophageal tumours, Stomach tumours, Colonic polyps, Colorectal cancers, Pancreatic and biliary tract cancers and Endocrine tumours of the GI tract respectively. In line with the editors specific chosen field of luminal cancers no attempt is made to cover the primary liver tumours although the management of hepatic secondaries from the GI tract is covered in the individual chapters.

The volume is well laid out and each chapter follows a clear uniform format covering epidemiology, aetiology, pathology, presentation, diagnosis, treatment and outcome. Where appropriate palliative surgical techniques and methods of pain relief are fully described as in the section on pancreatic cancer. Potentially confusing areas such as the differences between Duke's original staging of colorectal cancer and the subsequent more commonly used Astler-Coller and Kirklin-Dockerty modifications are clearly and concisely explained. Each chapter is individually referenced with 73 to 222 papers being cited. The references and the content of each chapter are fully up to date including for example the 1990 NIH consensus conference report on adjuvant chemotherapy and post operative radiotherapy for colorectal cancers.

The book is comprehensively illustrated to a very high standard. Histological sections and illustrations of specimens are beautifully photographed and are accompanied by useful explanatory line drawings where necessary. Radiographs, line drawings and the appearance of gross specimens are presented side by side. Schematic diagrams are used extensively to illustrate surgical techniques, lymphatic spread and tumour staging. All are clear and easy to follow as are the well presented tables and graphs.

As would be expected 'Gastrointestincal Cancer' coming from the Memorial Sloan-Kettering Cancer Center has a strongly American approach and reflects American practice and experience. British readers will note differences not only in the use of non SI units but also in the screening recommendations and therapeutic approach to some of the tumours described. Chemotherapy and radiotherapy are given greater prominence than would be usual UK practice but the evidence both for and against such approaches is included and well referenced so that the reader will be able to make his or her own judgement of the value of these treatment modalities.

Overall this is a very impressive book. The clear style and illustrations make it a pleasure to read and it will be a welcome addition to the shelves of physicians and surgeons in the field of gastroenterology and a popular volume in medical school libraries.

N.V. Jamieson